# Microenvironment immune response induced by tumor ferroptosis—the application of nanomedicine

**DOI:** 10.3389/fonc.2022.1019654

**Published:** 2022-09-16

**Authors:** Tian Yun, Zhenzhu Liu, Jianbo Wang, Rui Wang, Liang Zhu, Zheng Zhu, Xuejian Wang

**Affiliations:** ^1^Department of Urology, First Affiliated Hospital of Dalian Medical University, Dalian, China; ^2^Department of Cardiovascular, Second Affiliated Hospital of Dalian Medical University, Dalian, China; ^3^College of Basic Medical Science, Dalian Medical University, Dalian, China; ^4^Department of Medicine, Brigham and Women’s Hospital and Harvard Medical School, Boston, MA, United States

**Keywords:** ferroptosis, tumor microenvironment, nanotechnology, cancer therapy, immunity therapy

## Abstract

Ferroptosis is a non-apoptotic regulatory form of cell death that has sparked significant interest and research in cancer treatment and certain small chemical inducers have been used in the clinic. These inducers’s weak water solubility, poor targeting, rapid metabolism; and other undesirable characteristics; however, for therapeutic approaches that combine immunotherapy and ferroptosis, challenges such as medication delivery, the complexity of the tumor microenvironment, and immunosuppression remain. The targeted, low toxicity, and efficient distribution benefits of nanotechnology have considerably enhanced the therapeutic efficacy of combining immunotherapy with ferroptosis. This paper describes the distinct mechanism of ferroptosis in tumor therapy and immunotherapy, as well as the application and benefits of nanotechnology in the combination of tumor immunotherapy and ferroptosis.

**Systematic review registration:**

http://clinicaltrials.gov/, NCT00941070.

## Introduction

Ferroptosis is an iron-dependent form of nonapoptotic regulated cell death, characterized by the accumulation of lipid peroxides and production of reactive oxygen species (ROS) (1). Since ferroptosis was named in 2012, its mechanism and molecular pathways have been extensively studied, in which excessive accumulation of iron is one of the key steps, which is mainly caused by the Fenton reaction of excess ferrous iron to generate excessive ROS. On the other hand, when there is a lack of oxidative defense mechanisms inside the cell, such as inhibition of the Xc- system or GPX4, the accumulation of excessive ROS on the cell membrane exceeds the detoxification capacity, resulting in cell ferroptosis. Because ferroptosis is a multistep sequential mechanism that involves numerous pathways and intracellular organelles, there are multiple sites along this complex process where abnormalities have been linked to a variety of diseases, ranging from benign chronic conditions to cancer. Characterization of these sites has stimulated considerable research into therapeutic targeting especially for tumor treatment.

Emerging tumor immunotherapy has changed the way traditional chemotherapy fights tumors. The tumor microenvironment (TME) is comprised of complex cell types, including tumor cells, fibroblasts, immune cells, inflammatory cells, and others (2). These complex environments affect every aspect of the tumor. The relationship between various immune cells in the TME and ferroptosis has been extensively studied. In 2019, Wang et al. first demonstrated that CD8^+^T cells could trigger ferroptosis of tumor cell (3).

Nanoparticles (NPS) have the advantages of enhancing permeability and retention (EPR) in solid tumors and effectively transporting drugs or molecules targeting the interior of tumors, and have received extensive attention and research. For example, iron-based nanoparticles can rapidly release Fe^2+^Fe^3+^ into tumor cells, creating a high iron concentration environment, which can accelerate the production of ROS through the Fenton reaction thereby inducing ferroptosis (4). Furthermore, cancer treatment could be enhanced by combining chemotherapy with ferroptosis-mediated nanomedicines, such as iron oxide-coated NPS with cisplatin (5). The nanostructure helps the accumulation of cisplatin and iron in the tumor, the H_2_O_2_ released by cisplatin together with iron stimulates a Fenton reaction with formation of the hydroxyl radical -OH, which leads to ferroptosis of tumor cells.

This review provides an overview of the basic mechanisms of ferroptosis, explores the link between ferroptosis and immune cells in the TME, summarizes the findings of ferroptosis and tumor immunotherapy, discusses ferroptosis-based nanomedicines for effective cancer therapy, and discusses how to respond to ferroptosis. Conclusions, future research directions and challenges in developing nanomedicine-based ferroptosis and tumor immunotherapy into clinical treatments will be presented.

## The mechanisms of ferroptosis in cancer

**Figure 1:**
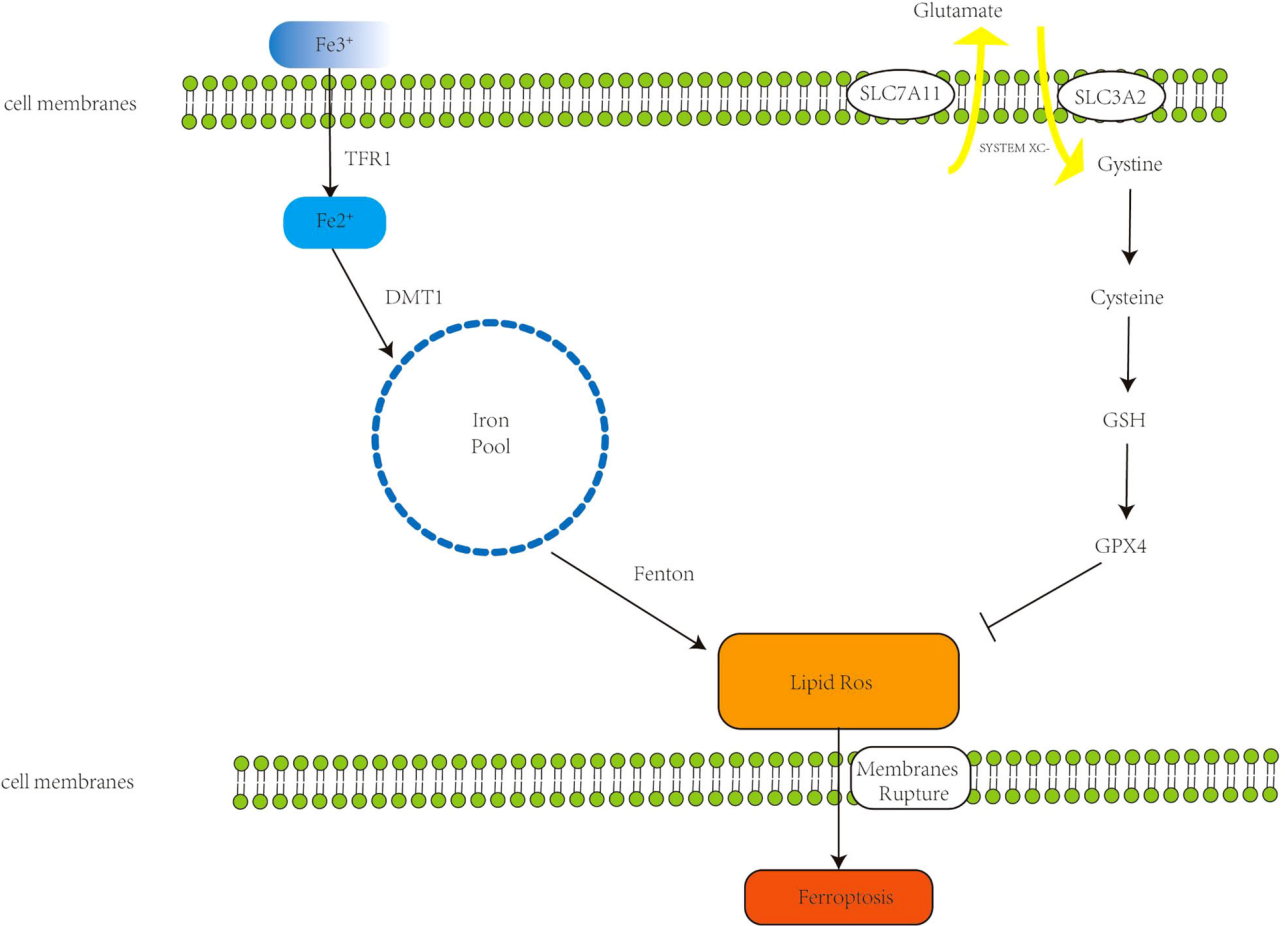
The primary mechanism of ferroptosis. Extracellular Fe^3+^ binds to transferrin receptor 1 (Tfr1) to form Fe^2+^; Fe^2+^ forms an unstable iron pool in the presence of divalent metal transporter 1 (DMT1). The Fenton reaction generates lipid reactive oxygen species (ROS), which cause cell membrane breakdown and, in the end, ferroptosis. The Xc- system, comprised of Recombinant Solute Carrier Family 3 (SLC7A11) and Recombinant Solute Carrier Family 7, Member 11 (SLC3A2), transports internal glutamate to the cell’s surface while also importing cystine, which initiates glutathione synthesis. Glutathione peroxidase 4 (GPX4) is a glutathione substrate that inhibits ROS and ferroptosis.

Since ferroptosis was proposed in 2012, its mechanisms and pathways have been extensively studied. There are three main mechanisms currently recognized for ferroptosis: iron accumulation, lipid peroxidation, and cell membrane rupture (**Figure 1**). Ferroptosis is closely related to the development of tumors. Because of its unique mechanism, ferroptosis is a potential target in cancer therapy (6).

### Accumulation of iron

Ferroptosis is an iron-catalyzed mode of programmed cell death. Increased iron absorption, reduced iron storage, and restricted iron efflux lead to increased iron accumulation, affecting intracellular iron concentration and promoting ferroptosis. Under normal conditions, iron exists in the form of ferric iron, which binds to transferrin receptor 1 (TFR1) and enters the endosome through the cell membrane, and then ferric iron is reduced to ferrous ions by ferroreductase catalyzed reaction (-ferrous iron) (7). Under the action of divalent metal transporter 1, ferrous iron reaches the cytoplasm through the endosomal membrane, forming an unstable iron pool. The accumulated excess iron can generate ROS through the Fenton reaction [the Fenton reaction has been described as follows: Fe^2+^ +H_2_O_2_ = Fe^3+^ + ·OH + HO^-^, Fe^3+^ + H_2_O_2_ = Fe^2+^ + ·OOH + H^+^ (8)] leading to ferroptosis (9). Dixon et al. experimentally demonstrated that deferoxamine (DFO), an iron chelator, can target the Fenton reaction to reduce ROS production and inhibit ferroptosis (1). Intracellular excess Fe^2+^ is usually stored in the form of ferritin, which contains two subunits, FTH1 (ferritin heavy chain 1) and FTL (ferritin light chain) (10). FTH1 functions to store iron. Downregulation of FTH1 increases Fe^2+^ concentration and promotes ferroptosis (11). Ferroportin (FPN), also called SLC11A3, inhibits ferroptosis by oxidizing ferrous iron to ferric iron for export out of the cell. Downregulation of FPN maintains high intracellular iron concentrations and sensitizes cells to ferroptosis (12). Therefore, increasing iron accumulation is a key step in the induction of ferroptosis.

### Lipid peroxidation

Ferroptosis mainly causes the accumulation of iron-dependent lipid peroxides, which are biomarkers of ferroptosis (13). Polyunsaturated fatty acids (PUFAs) contain bis-allylic hydrogen atoms, which are easily oxidized by ROS to lipid peroxides and promote ferroptosis. Acyl-CoA synthetic long chain family member 4 (ACSL4) and lysophosphatidylchoyltransferase (LPCAT3) incorporate PFUA activation into membrane PL (phospholipids) leading to ferroptosis (14). ACSL4 is a key factor in controlling cellular sensitivity to ferroptosis induction by regulating ferroptotic lipids (15). Inactivation of ACSL4 during apoptosis may inhibit PUFA insertion into the membrane, thereby limiting the ability of cells to undergo ferroptosis (16), and inhibition of ACSL4 or LPCAT3 inhibits ferroptosis by reducing lipid peroxides (17, 18). Additionally, P450 oxidoreductase (POR) induces ferroptosis by promoting lipid peroxidation (19).

However, under normal physiological conditions, iron-mediated lipid oxidation is counteracted by the cellular antioxidant system (20). The Xc-system and glutathione peroxidase (GPX4) are key regulatory targets of lipid peroxides. The Xc- system is a cystine-glutamate transporter composed of disulfide-linked heterodimers SLC7A11 and SLC3A2, which mediates transport of intracellular glutamate to the outside of the cell, and simultaneously imports cystine into the cell which generates synthesis of glutathione (GSH) (21). Thus, selective inhibition of the Xc- system reduces GSH synthesis, leading to excessive accumulation of toxic lipid ROS and promoting ferroptosis (22). GPX4 is the principal regulator in the antioxidant reduction reaction, which can reduce lipid peroxides to corresponding alcohols or H_2_O_2_ to water (23, 24); these properties of GPX4 establish it as a key factor inhibiting ferroptosis in cancer cells. When GPX4 is inhibited, lipid peroxides gradually accumulate, through a reaction catalyzed by intracellular iron, leading to cell death (ferroptosis) (25–27). The substrate of GPX4, GSH, is an antioxidant. Inhibiting System xc- inhibits the absorption of cystine and affects the synthesis of GSH (28), which in turn leads to the reduction of GPX4 activity, that reduces cellular antioxidant capacity, thereby promoting ferroptosis (29). There are small-molecule drugs such as erastin and sorafenib that reportedly can target the Xc-system, ultimately promoting ferroptosis (30, 31). In addition, Hassannia et al. demonstrated that withaferin A, a steroidal lactone derived from a medicinal plant, can directly target GPX4 to promote ferroptosis (32).

### Cell membrane changes

Ferroptosis is a novel form of regulated cell death (RCD) in which iron-catalyzed accumulation of polyunsaturated fatty acid phospholipids (PUFA-PLs) and toxic peroxidation in cell membranes reach lethal levels (33, 34). Lipids maintain the integrity of cell membranes, and when the accumulation of lipid peroxides on the cell membrane exceeds the detoxification capacity of antioxidant defenses, it will cause cell membrane to rupture, and the cell will undergo ferroptosis (35). ACSL4 enriches cell membranes with long polyunsaturated omega 6 fatty acids that are susceptible to ferroptosis, and inactivation of ACSL4 may inhibit PUFA insertion into the membrane, thereby limiting the ability of cells to undergo ferroptosis. When cells undergo ferroptosis, mitochondria undergo condensation with reduction in mitochondrial cristae that are depicted morphologically (22), which is distinct from typical apoptotic features.

## Ferroptosis and TME

Although current cancer chemotherapy has achieved indisputable advances, the complex dynamic environment inside the tumor can eventually develop drug resistance, and the chemotherapeutic drugs often have associated significant side effects. In recent years, immunotherapy has evolved as a novel modality for cancer treatment. The tumor microenvironment (TME) interacts with cancer cells leading to tumor growth, progression and drug resistance. The TME consists of cancer-associated fibroblasts (CAFs), endothelial cells, immune cells, inflammatory cells, among others, inside the tumor (36). In 2019, Wang et al. demonstrated for the first time that CD8^+^ T cells could drive ferroptosis in tumor cells, a pioneering experiment that added to the growing number of studies on ferroptosis and immunotherapy (3).

### Ferroptosis and T cells in the TME

Immune cells in the TME principally include T cells, macrophages, dendritic cells, B cells and NK cells. CD8^+^ directly fights tumors by releasing perforin, and Th1 secretes IFN-γ, TNF-α, etc. A variety of cytokines mediate anti-tumor immune responses (37). In ferroptosis, CD8^+^ T cells release interferon γ (IFNγ) to downregulate the expression of two subunits of system Xc-, SLC3A2 and SLC7A11, which inhibit the uptake of cystine by tumor cells, resulting in cysteine depletion, with consequent glutathione depletion, and impaired GPX4 activity, thereby promoting ferroptosis in tumor cells; therefore, CD8^+^ T cells contribute to the antitumor effect of immunotherapy (3). Notably, the high-fat environment within the TME overexpressed the fatty acid transporter CD36, which in turn enhanced the sensitivity of CD8^+^ T cells to ferroptosis and weakened the antitumor effect of the CD8^+^ T cells (38).

Tregs (regulatory T cells) are derived from CD4^+^ T cells, normally suppress abnormal or excessive immune responses (39), and are also involved in promoting immune escape and maintaining tumor growth. Therefore, an increased proportion of Treg cells in tumors usually predicts a poor prognosis in cancer patients. Inhibition of Treg cell survival in the TME has emerged as a new strategy to enhance tumor immunotherapy. The hyperoxidative environment in the TME reportedly induces Treg cells to exhibit enhanced immunosuppressive function (40). GPX4 was introduced previously using GSH as a cofactor to reduce lipid peroxidation, and inactivation of GPX4 can induce ferroptosis. Inhibition or depletion of GPX4 induces ferroptosis in Treg cells within tumors and enhances the antitumor immune response (41).

### Ferroptosis and macrophages in the TME

Macrophages in the TME (TAMs) are involved in every stage of cancer from proliferation to invasion to metastasis (42). M1M2 are two activation states of TAMs: M1 macrophages (pro-inflammatory phenotype) and M2 macrophages (anti-inflammatory phenotype) (43). Early in tumor development, M1 macrophages can destroy tumor cells, whereas during tumor progression, the TME begins to favor the transition of macrophages to the immunosuppressive and tumor-promoting M2 type (44). Therefore, targeting M2-type TAMs to deplete or revert M2-type to M1-type in the TME, enhancing their cytotoxicity and indirectly stimulating cytotoxic T cells, serves as a potential strategy for antitumor immunotherapy. M1 macrophages have been shown to be more resistant to drug-induced ferroptotic death than M2 *in vivo* depletion of GPX4 in TAMs can inhibit survival of M2-type macrophages without affecting the number of M1-type macrophages (45). According to Jiang et al., nanoparticles can not only induce ferroptosis by inhibiting the glutamate-cystine anti-transport system XC-, but also effectively repolarize M2 phenotype macrophages to M1 phenotype (46). However, ferroptotic cancer cells can release KRAS-G12D protein, induce macrophages to switch to M2 type, and promote tumor progression (47).

### Ferroptosis and other immune cells in the TME

Natural killer (NK) cells are central to antitumor immunity and have recently demonstrated efficacy against malignant tumors (48). While NK cells in the TME are dysfunctional due to lipid peroxidation inhibiting glucose metabolism, some ferroptosis inhibitors can restore the glucose metabolism and antitumor ability of NK cells *in vivo* (49). However, ferroptotic cancer cells release PGE2 could damage the anti-tumor immunity of the immune system by targeting NK cells, which promoting the progression of colon cancer (50).

Dendritic cells (DC) are antigen-presenting cells (APCs). Under normal circumstances, DCs exist in an immature form and play a key role in anti-tumor immunity by activating toxic T cells (51). Both the (peroxisome proliferator-activated receptor δ) PPARG-dependent ferroptosis antigen presentation ability and the ability to activate T cells have been found to be impaired in DCs, so the combined action of ferroptosis activators and PPARG inhibitors may enhance anti-tumor effect and reduce side effects (52).

**Figure 2:**
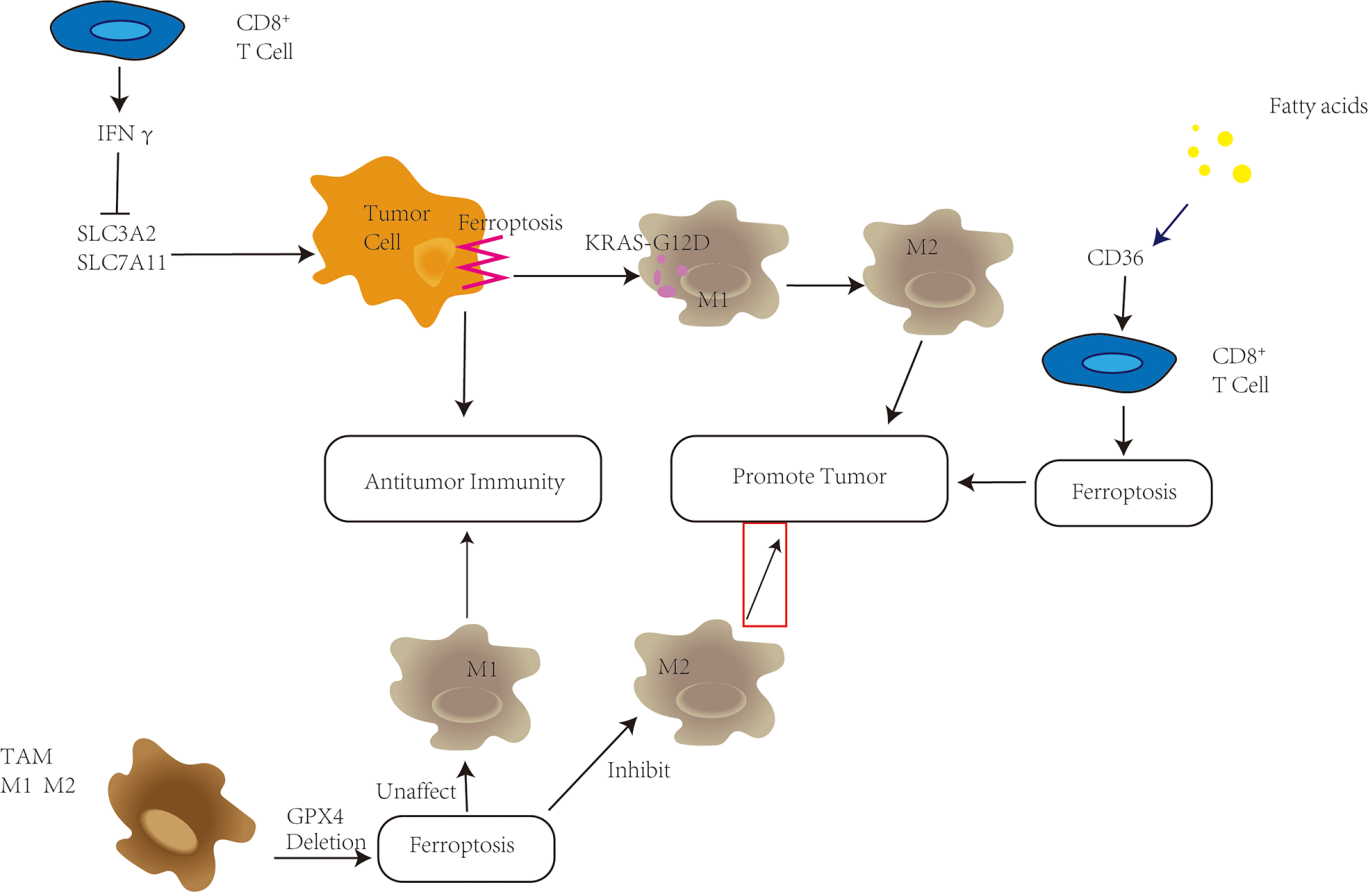
Ferroptosis plays a variety of roles in the tumor microenvironment (TME). CD8^+^ T cells in the TME have been shown to secrete IFNγ, which inhibits SLC3A2 and SLC7A11, promotes tumor cell ferroptosis, and improves antitumor immunity. High fatty acid levels in the TME, on the other hand, enhance CD36 upregulation, trigger ferroptosis in CD8^+^ T cells, and hasten tumor growth. Depletion of GPX4 in tumor-associated macrophages (TAMs) can reduce M2-type macrophage survival while increasing antitumor immunity without changing the amount of M1-type macrophages. KRAS-G12D is released pancreatic cells undergo ferroptosis, promoting the transition of macrophages into M2 type and tumor development.

According to the above, ferroptosis can have dichotomous actions related to the TME: it can either enhance the effect of immunotherapy or promote tumor survival and progression The key to clinical application of ferroptosis would be to design a safe and effective process (**Figure 2**)

ICB refers to enhancing the antitumor activity of antigen-specific T cells and releasing endogenous anti-tumor immune responses. Currently of high concern ICB treatments include Cytotoxic T-lymphocyte antigen 4 (CTLA-4) and Programmed cell death protein 1 (PD-1) or programmed death-ligand 1 (PD-L1). According to the above experiments of Wang et al., it was found that the use of PD-L1 blocked immune checkpoint therapy can enhance T cell-mediated anti-tumor immunity and induce ferroptosis in tumor cells. These findings confirm that immunotherapy can work in part by promoting ferroptosis in tumors. While T cells in the TME are at the heart of immunotherapy, studies have revealed that some innate immune cells, such as macrophages and NK cells, also have an effect on the ICB.

## Applications of nanotechnology in ferroptosis and tumor immunity

**Figure 3:**
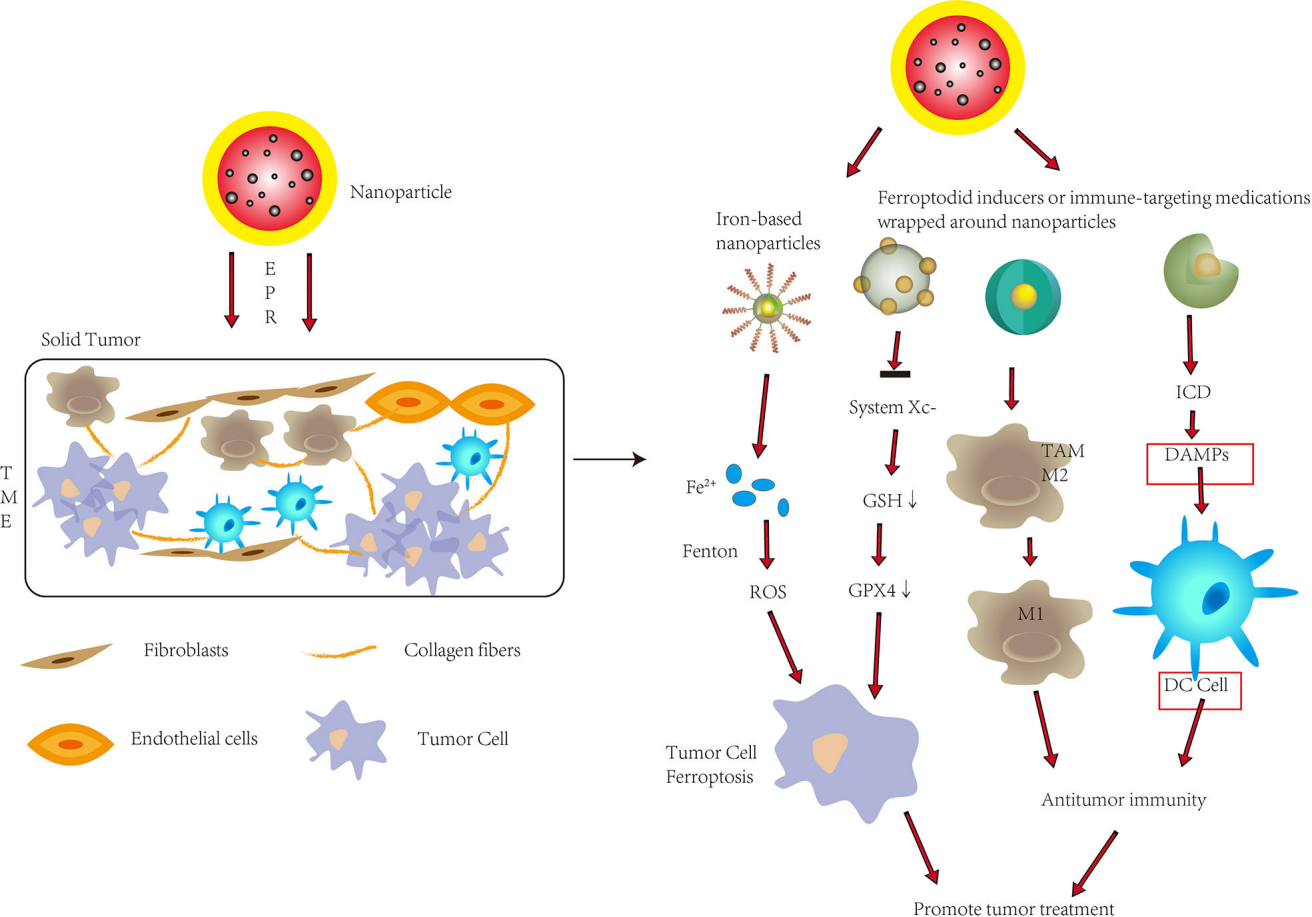
To improve tumor therapy, nanoparticles are used in ferroptosis and tumor immunity. Nanoparticles inside tumors can promote ferroptosis in tumor cells by releasing significant levels of Fe^2+^ or suppressing System Xc-, allowing them to penetrate solid tumors through enhanced permeability and retention (EPR). Nanoparticles can also convert M2-type cells to M1-type cells inside tumors, improve immunogenic cell death (ICD), and promote DC cell maturation, all of which improve anti-tumor immunity. To improve tumor therapy, nanoparticles can trigger ferroptosis while also increasing antitumor immunity in solid tumors. DAMPs, Damage associated molecular patterns.

Nanotechnology has been rapidly developed and widely used in the medical field. The application of includes (1) drug loading of nanoparticles (NPs) (2) direct use of nanoparticles as drugs (3) both drug loading and drug use. Its targeting, low toxicity, and the advantages of extended permeability and retention (EPR) in tumors have attracted great attention and research in both ferroptosis and tumor immunotherapy (**Figure 3**)

### Nanotechnology in ferroptosis

Traditional biological preparations of small molecule ferroptosis inducers usually have the disadvantages of poor water solubility, too rapid metabolism in the body, and low targeting ability, which compromise the effects of inducing ferroptosis. Therefore, the use of nanotechnology can improve the delivery, release and targeting efficiency of drugs to enhance the induction of ferroptosis. The use of nanotechnology to deliver targeted ferroptosis drugs has been extensively studied in recent years. For example, Yang et al. synthesized monodispersed ferrihydrite nanoparticles (PEG-Fns) with a good uptake rate and compatibility at tumor sites (53). Under ordinary blue light irradiation, considerable Fe^2+^ was released, which promoted the production of ROS and led to ferroptosis. Nanotechnology can also be used to endow nanomaterials with special functions. For example, Tang et al. designed targeting sorafenib (SFB)-loaded manganese-doped silica nanoparticles (FaPEG-MnMSN@SFB) (54). Degradation of the MnMSN consumed intracellular GSH and together with SFB inhibited the XC- system, which disrupted the balance of intracellular redox, and promoted ferroptosis. Similarly, miR-101-3p, a microRNA that functions as a tumor suppressor in several types of cancer, can increase ROS levels in cells, reduce GSH levels, induce ferroptosis, which inhibits cancer cell proliferation. However, it is known that nucleic acids, including miRNAs (such as miR-101-3p) cannot be directly treated *in vivo* by commonly used intravenous or oral administration. Therefore, Luo et al. used nanocarriers to achieve *in vivo* delivery and therapeutic effects of miR-101-3p (55). In addition, the Fe_3_O_4_-SAS@PLT designed by Jiang et al. was constructed from sulfasalazine (SAS)-loaded mesoporous magnetic nanoparticles (Fe_3_O_4_) and platelet (PLT) membrane camouflage, which could evade immunity (46). This complex MSN could take advantage of the innate characteristics of platelets to quickly identify vascular damage and identify and aggregate with circulating tumor cells. Fe_3_O_4_ can increase iron abundance, leading to the Fenton reaction to generate ROS; SAS can inhibit cysteine transport, lead to ferroptosis, and can effectively repolarize macrophages from M2 phenotype to M1 phenotype. The combination of nanotechnology and ferroptosis can more effectively improve the anti-tumor therapeutic effect of ferroptosis and avoid the limitations of small molecule ferroptosis inducers.

### Nanotechnology in immunotherapy

Although tumor immunotherapy has achieved substantial clinical success, most patients still cannot benefit from current immunotherapy and may experience serious side effects. High toxicity is a problem with immunotherapy medications, however, nanotechnology can improve immune responses and lessen extratumor stimulation. For instance, encapsulating immunological medicines in liposomes can decrease their systemic toxicity while also shortening their half-life in the blood. Meanwhile, using nanotechnology to target immune suppressive cell types in the TME could improve immunotherapy.

The tumor microenvironment (TME) poses a major obstacle to immunotherapy, not only affecting drug delivery but also leading to immunosuppression. Through current study, the use of nanotechnology can enhance immune regulation by integrating various molecules, facilitate targeted delivery to manipulate immune cells, and enhance the effect of immunotherapy. Drug encapsulation within nanoparticles extends *in vivo* circulation time, reduces off-targets, and enables synergistic encapsulation of multiple drugs into a single nanoparticle. Nanomaterials take advantage of preferentially accumulating in solid tumors due to abnormally leaky vasculature and dysfunctional lymphatic drainage within the TME—a phenomenon known as enhanced permeability and retention (EPR) (56). When targeting cancer cells, nanomedicines mainly induce specific immune responses by inducing immunogenic cell death (ICD) and releasing immunostimulatory damage-associated molecular patterns (DAMPs), such as calreticulin and high mobility group box 1(HMGb1) (6, 57). ICD is induced by some chemotherapeutic drugs, radiation therapy, photodynamic therapy, among others (58). Zhao et al. encapsulated oxaliplatin (OXA) into amphiphilic diblock copolymer nanoparticles, that enhanced ICD through nanoparticle delivery, and *in vitro* NP encapsulation enhanced the ability of OXA to induce DAMPs and dendritic cells maturation to achieve a more powerful immune response from cytoplasmic and T cells (59).

When targeting the tumor immune microenvironment, nanomedicines enhance immunotherapy by suppressing immunosuppressive cells and reducing immunosuppressive molecules. Wang et al. developed an assembly of an exosome inhibitor (GW4869 is a cell-permeable, non-competitive neutral sphingomyelinase inhibitor; an exosome inhibitor that blocks ceramide-mediated multivesicular body sprouting and thereby inhibits multivesicular body sprouting exosome) and a ferroptosis inducer (Fe^3+^) *via* amphiphilic hyaluronic acid, the released GW4869 significantly decreased tumor-derived exosome generation, to induce antitumor immunity and stimulate cytotoxic T lymphocytes (60). Furthermore, Guo et al. designed a gel delivery, system incorporating embedded gold nanorods (AuNRs) and iron oxide nanoparticles (IONs) for bladder cancer treatment (61). *In vitro* experiments verified that TAMs of M2 phenotype could be repolarized by these IONs to TAMs of M1 phenotype to exert antitumor effects. Evidence to support this demonstrated long protrusions that were round and flat with spicules on the edges, typical of transformation towards the M1-like phenotype. Additionally, the M2-like macrophages were then examined at the transcriptional level to upregulate CD80 and downregulate CD206, indicating M2 to M1 transformation. Finally, ELISA showed that ION-treated macrophages exhibited increased secretion of IL-1β, IL-12 and TNF-α markers of M1. Subsequent *in vivo* experiments using immunofluorescence to examine day-5 tumor tissue of an intravesical drug-treated mouse model of balloon bladder cancer (APBCa), found that treatment with embedded IONS produced increased CD80, indicating transformation of M2-like macrophages into M1.

In conclusion, nanotechnology can enhance tumor immunotherapy in a variety of ways, some of which have been tested in humans. The future seems bright for nanotechnology to provide substantial advances in clinical diagnosis and treatment.

## Conclusion and outlook

Current conventional chemotherapy for tumors. which frequently results in drug resistance and systemic side effects, resulting in either a suboptimal or unsuccessful outcome. Therefore, new treatment modalities need to be developed. Ferroptosis is an iron-dependent form of programmed cell death mainly characterized by iron accumulation, lipid peroxidation, and changes in cell membranes. The discovery of ferroptosis prompted new directions for cancer treatment. There is abundant evidence that ferroptosis inducers can effectively kill tumors and overcome drug resistance. In addition, ferroptosis has now been used synergistically with various conventional and novel treatment options in cancer, including chemotherapy, radiotherapy, and immunotherapy. Of particular emphasis, the combination of ferroptosis and immunotherapy has attracted great attention. By activating T cells in the TME, polarizing the immunosuppressive M2 phenotype macrophages to the anti-tumor M1 phenotype, and suppressing immunosuppressive factors dramatic research interest has been stimulated. Nevertheless, whether ferroptosis or immunotherapy have been used alone or in combination shortcomings and deficiencies have been encountered. The use of nanotechnology has very reasonable prospects of reducing these issues. Nanotechnology can not only enhance the efficiency and cycle life of ferroptosis inducers, but also target specific immune cells in the TME. In addition, nanomedicine is an ideal drug delivery tool, which can encapsulate a variety of drugs in a tiny particle.

Of course, there are inevitably questions that will require further investigation to answer. What is the best combination using nanotechnology to target ferroptosis with immunotherapy? Safe, non-toxic nanomaterials must be designed and tested *in vivo*, and degradation of nanomaterials must be included in the process of *in vivo* evaluation. Nanotechnology has provided exciting opportunities and directions for the treatment of malignancies, a field that will no doubt continue to expand and achieve breakthroughs in cancer treatment.

## Data availability statement

The original contributions presented in the study are included in the article/supplementary material. Further inquiries can be directed to the corresponding authors.

## Author contributions

YT: conceptualization, methodology, investigation, formal analysis, and writing - original draft. LZ: data curation and writing - original draft. JW: visualization and investigation. WR: resources and supervision. LZ: software and validation. ZZ: visualization and writing - review & editing. XW: conceptualization, funding acquisition, resources, supervision, and writing - review & editing. All authors contributed to the article and approved the submitted version.

## Funding

This research was supported by the grant from a National Natural Science Foundation of China (No.31700818 and No.82173121).

## Conflict of interest

The authors declare that the research was conducted in the absence of any commercial or financial relationships that could be construed as a potential conflict of interest.

## Publisher’s note

All claims expressed in this article are solely those of the authors and do not necessarily represent those of their affiliated organizations, or those of the publisher, the editors and the reviewers. Any product that may be evaluated in this article, or claim that may be made by its manufacturer, is not guaranteed or endorsed by the publisher.
